# Advanced configuration of hybrid passive filter for reactive power and harmonic compensation

**DOI:** 10.1186/s40064-016-2917-7

**Published:** 2016-08-02

**Authors:** O. Fatih Kececioglu, Hakan Acikgoz, Mustafa Sekkeli

**Affiliations:** 1 Department of Electrical and Electronics, Faculty of Engineering, Kahramanmaras Sutcu Imam University, Kahramanmaras, Turkey; 2 Department of Electrical Science, Kilis 7 Aralik University, Kilis, Turkey

**Keywords:** Hybrid passive filters, Harmonics, Power quality, Reactive power compensation

## Abstract

Harmonics is one of the major power quality problems for power systems. The harmonics can be eliminated by power filters such as passive, active, and hybrid. In this study, a new passive filter configuration has been improved in addition to the existing passive filter configurations. Conventional hybrid passive filters are not successful to compensate rapidly changing reactive power demand. The proposed configure are capable of compensating both harmonics and reactive power at the same time. Simulation results show that performance of reactive power and harmonic compensation with advanced hybrid passive filter is better than conventional hybrid passive filters.

## Background

In the latest years, harmonic distortion has become one of the most significant power quality problems. The primary causes of this problem can be sorted as soft starters, rectifiers and increase of devices that of semiconductor circuits. Nonlinear loads cause harmonic distortion within the voltage and current waveform in the power system. Harmonics result in numerous problems such as low power factor and overheat on the power systems, electrical devices and transformers (Lee and Wu 1998; Snal et al. 2004; Hamadi et al. 2010; Sekkeli and Tarkan 2013). In order to protect other users in power system from the effects of the harmonics caused by nonlinear devices, the IEEE 519-1992 standard has imposed specific limits on levels of voltage and current harmonics. Mainly, it sets limits of harmonic current and voltage at the point of common coupling.

Harmonic distortion has been suppressed by passive filters, active filters, and hybrid filters. Among these, the passive filters have been widely applied in filtering harmonics in power systems up to the present since it has high reliability, efficiency, low cost and a simple configuration. Also, passive filters are preferred where harmonics and reactive power compensation have been desired. Many different topologies of passive filters have been suggested in the literature, and the parallel filter configuration is most preferred filter topologies (Thirumoorthi and Yadaiah 2015; Zobaa 2005; Singh and Verma 2007; Cheng et al. 1996).

Parallel passive filters are more suitable for compensating current source nonlinear loads. On the other hand, it has been shown that the parallel passive filter is suitable for compensating current source type of nonlinear loads. The series passive filter can be used to compensate for voltage source type of nonlinear loads. Hybrid passive filter (HPF) which consist of a serial passive filter and parallel passive filter can be used for all type of nonlinear loads. The HPF delivers harmonic and reactive power compensation and is also insensitive to source impedance (Prasad and Sudhakar 2014; Dzhankhotov and Pyrhonen 2013; Jou et al. 2001).

Despite the fact that HPF is considerable performed to harmonic mitigation, this filter cannot be fully successful to compensate the reactive power for suddenly changing nonlinear loads.

In the study by Rahmani et al. proposed a new single phase hybrid passive filter (SPHPF) for compensating load voltage and current harmonics, correct power factor. Additionally, the SPHPF eliminate the chances of series and parallel resonance and eliminates large variation of power factor and terminal voltage with varying loads under stiff and distorted source conditions (Rahmani et al. 2008).

Singh et al. focused on new hybrid passive filter topology, which provides harmonic compensation at par with active filters, whose design is insensitive to source impedance, eliminate the chances of resonance over wide spectra and reduces large variation of power factor and terminal voltage with varying rectifier load (Singh et al. 2005).

In the study by Hsan et al. proposed a shunt hybrid power filter (SHPF) which consists of a small-rated active power filter in series with a fifth-tuned passive filter. Since the latter takes care of the major burden of compensation, the rating of the shunt hybrid power filter is much smaller than that in the conventional shunt active power filter (Hsan et al. 2013).

Hamadi et al. proposed a novel topology for a three phase hybrid passive filter (HPF) to compensate for reactive power and harmonics. The proposed HPF configuration has many features such as: insensitivity to source-impedance variations; no series or parallel resonance problems; fast dynamic response. According to experimental and simulation results show that the proposed HPF configuration provides compensate all voltage and current harmonics and reactive power for large nonlinear loads (Hamadi et al. 2010).

Few researchers have investigated the hybrid passive filter configuration in order to compensate for reactive power and harmonics (Hamadi et al. 2010; Rahmani et al. 2007). The performance of the hybrid passive filter has been investigated for any load types such as rectifiers and motor drivers. Despite that, the nonlinear loads are acceptable for harmonics mitigation performance of the filters, these loads are not suitable for reactive power compensation performance. Since reactive power demand of the loads has been minimized. In order to analysis of reactive power compensation performance of filter should be used varying loads or suddenly switched on/off loads.

This paper proposes a new configuration of hybrid passive power filter in order to overcome the above-mentioned harmonic standard. The advanced hybrid passive filter (AHPF) configuration is composed of two thyristor controlled parallel passive filters (TCPF) and a serial passive filter (SPF). The AHPF is designed to rapidly changing nonlinear loads in order to reactive power compensation. The TCPF is capable both reactive power compensation and current harmonics mitigation of nonlinear loads.

This paper is arranged as follows: “Hybrid passive filters” section briefly presents theory of hybrid passive filter. “Advanced hybrid passive filter configuration” section presents details of advanced hybrid passive power filter. Simulation studies and results of AHPF shows in “Simulation results” section. “Conclusion” section delivers our conclusions and a brief discourse on future research directions.

## Hybrid passive filters

**Fig. 1:**
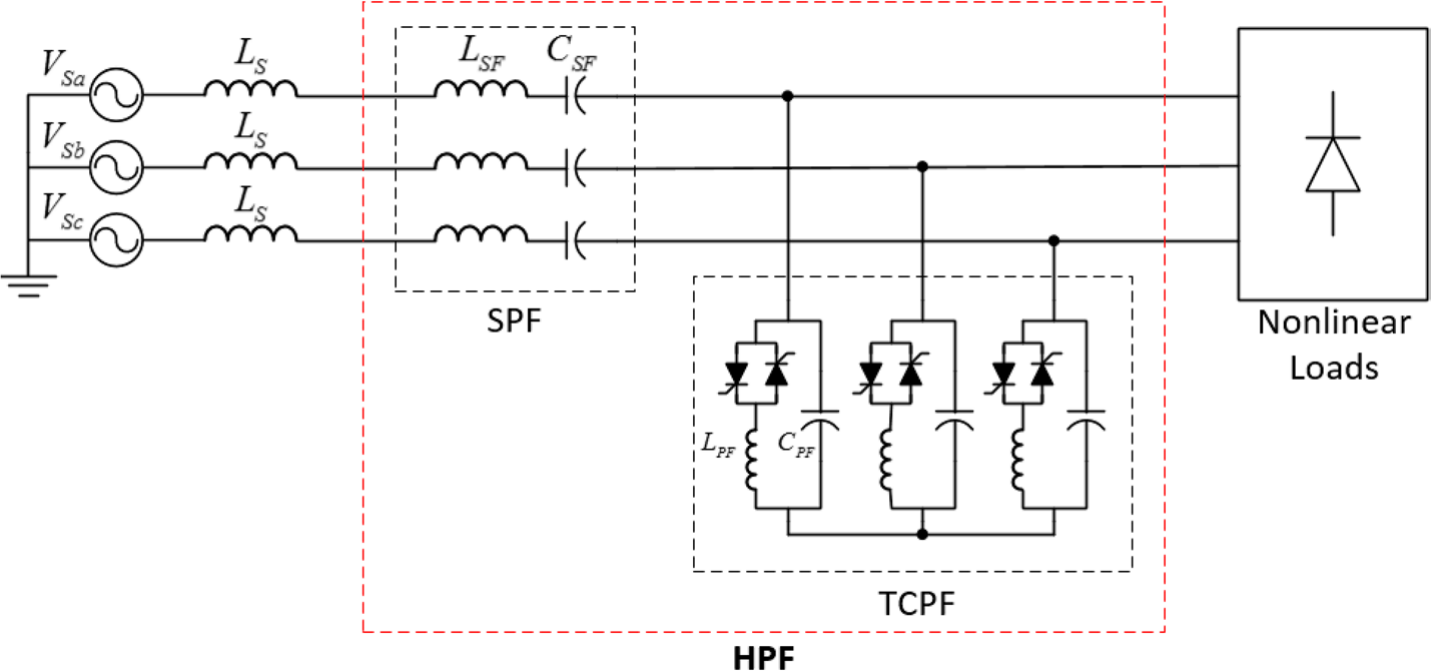
Configuration of hybrid passive filter

Conventional HPF configuration that is composed of a TCPF and a SPF is illustrated in Fig. 1. In this configuration, the SPF and TCPF operate as a bandpass filter and a bandstop filter respectively. The HPF is connected to between the nonlinear loads which produce voltage and current types of harmonics and point of common coupling (PCC) in the power system.

The SPF is presented a low impedance at the fundamental frequency thus absorbing the voltage harmonics of interest. While SPF blocks for voltage fed type of harmonics, the TCPF eliminates to current fed type harmonics. Therefore, HPF is able to compensate to all type of harmonics caused by nonlinear loads (Rahmani et al. 2007).

### Series passive filter

**Fig. 2:**
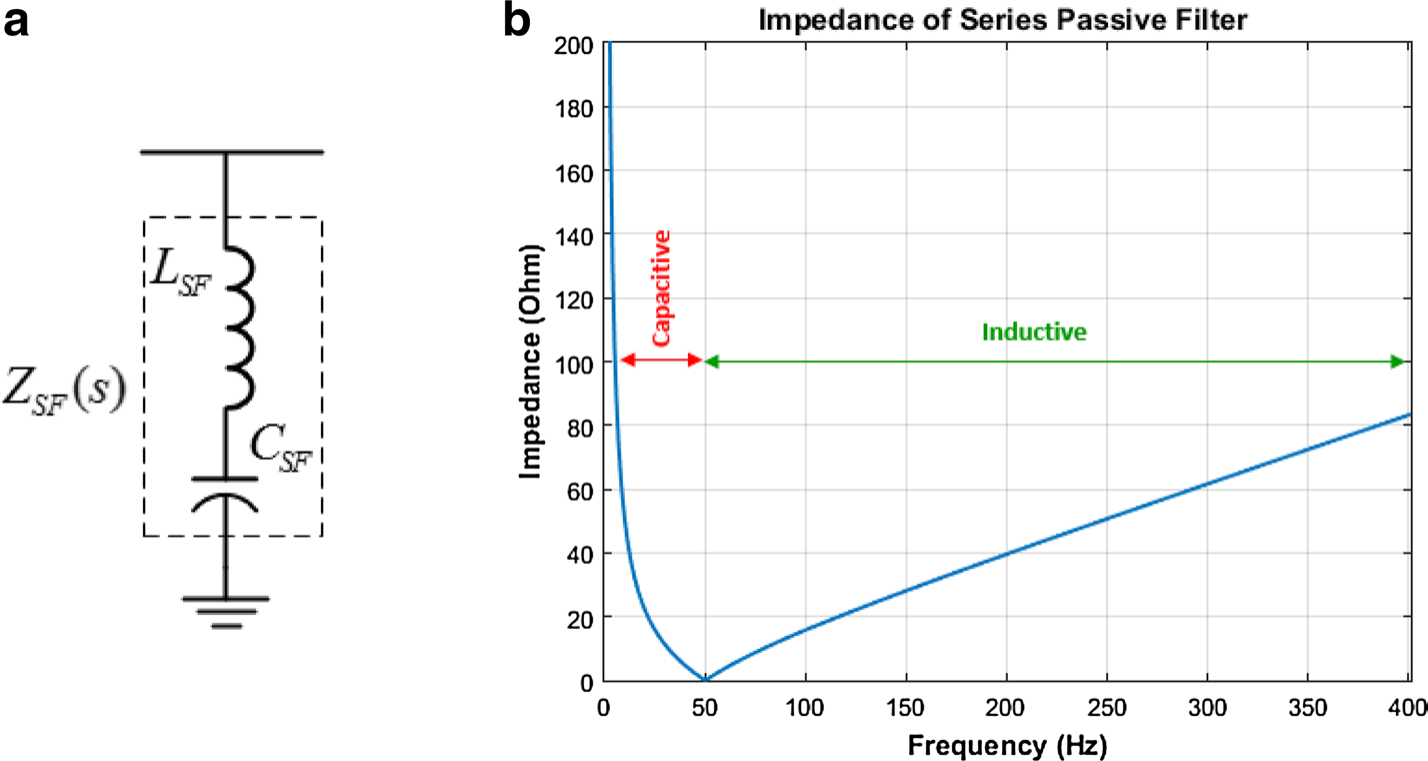
Single phase equivalent circuit (**a**) and frequency response (**b**) of series passive filter

Series passive filter is consist of series connection of a capacitor and a reactor. The SPF blocks flow of the current type harmonics in the direction of the source side by supplying high impedance path at all harmonic frequencies. At the fundamental frequency, the capacitor and reactor have equal impedance. Resonant frequency of SPF is selected at a value close to the power system frequency. Single phase equivalent circuit of SPF and impedance response is shown in Fig. 2. Impedance response of series passive filter is expressed as a transfer function. The transfer function is calculated for a single-phase equivalent circuit. This transfer function is defined as (Hamadi et al. 2010; Phipps 1997);

1 $$ H_{F} (s) = Z_{SF} (s) = \frac{{s^{2} (L_{S} C_{SF} + L_{SF} C_{SF} ) + 1}}{{sC_{SF} }} $$

Figure 2b illustrate that the SPF offers high impedance to all higher harmonic frequencies. Concurrently, SPF presents very low impedance at the fundamental frequency. This is significant because notable impedance at the network frequency may lead to considerable voltage drop.

### Thyristor controlled passive filter

**Fig. 3:**
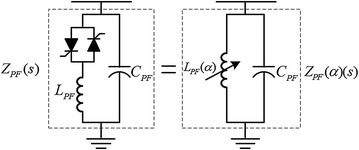
Equivalent circuit of TCPF

TCPF consists of a reactor, a capacitor and thyristor valve. The TCPF offers high impedance at the fundamental frequency, however, presents low impedance for all higher harmonic frequencies. The single phase equivalent circuit of TCPF is illustrated in Fig. 3.

The TCPF supplies a low impedance sink for currents at harmonic frequencies to prevent the flow of harmonics towards PCC. The filter’s characteristics are capacitive for all higher harmonics and inductive for below the fundamental frequency. The output impedance transfer function of TCPF is defined as,

2 $$ Z_{PF} (\alpha )(s) = H_{f} (s) = \frac{{sL_{PF} (\alpha )}}{{s^{2} L_{PF} (\alpha )C_{PF} + 1}} $$

The equivalent inductance of the star connection is given by (Garcia-Cerrada et al. 2000; Alves et al. 2008),

3 $$ L_{PF} (\alpha ) = L_{PF} \frac{\pi }{2\pi - 2\alpha + \sin (2\alpha )} $$

where the firing angle is bounded as (*π*/2) < *α* < *π*.

**Fig. 4:**
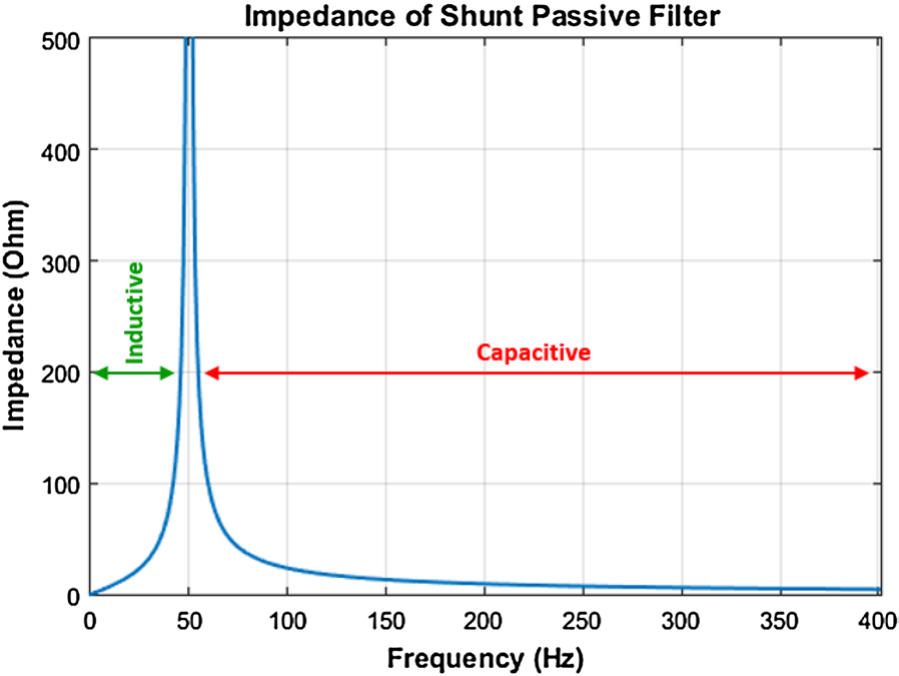
Frequency response of TCPF

The impedance response of TCPF that is triggered *π*/2 firing angle is shown in Fig. 4. The TCPF offers low impedance path for all harmonics currents, therefore, protecting against the harmonics to flow through the source while preventing the fundamental current from following into the TCPF.

## Advanced hybrid passive filter configuration

**Fig. 5:**
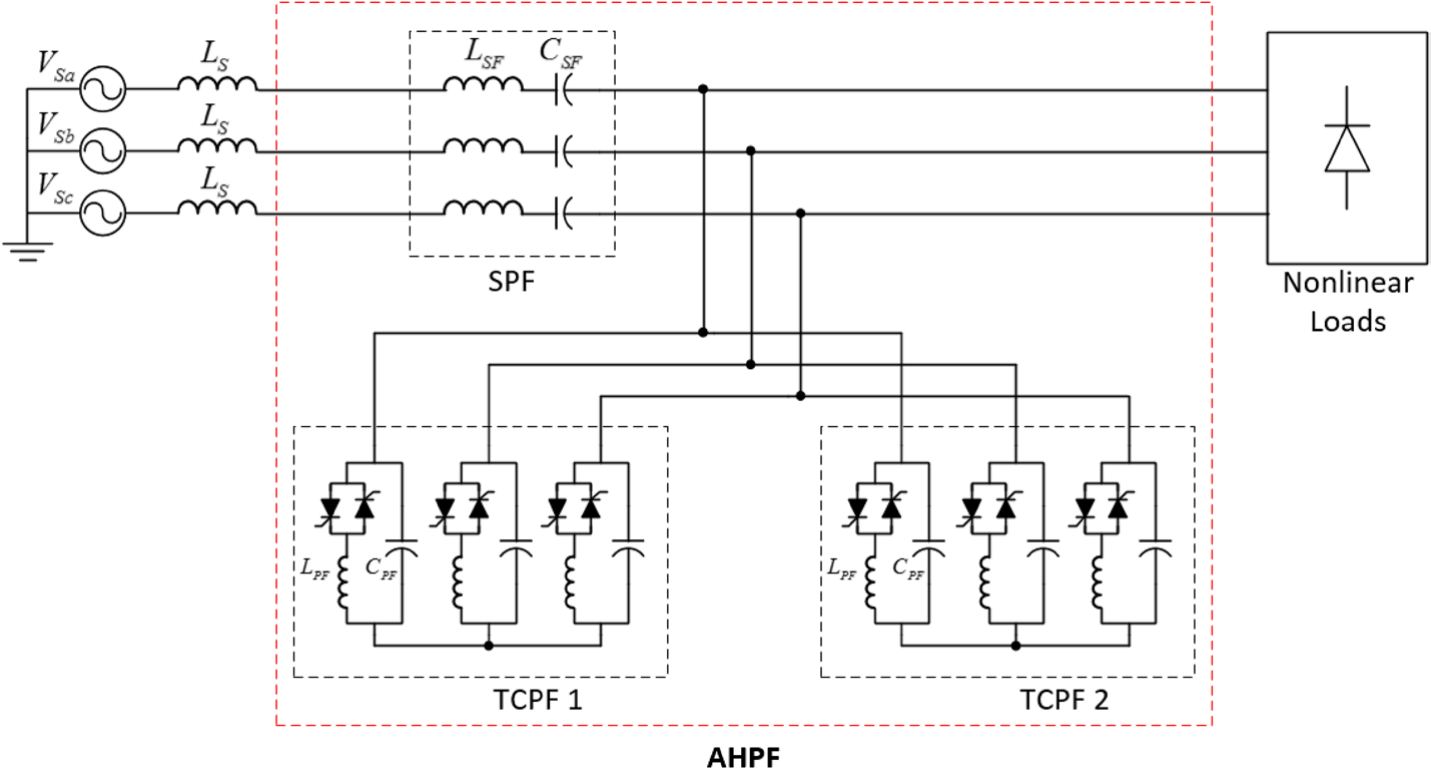
Configuration of AHPF

Proposed AHPF system configuration is shown in Fig. 5. The AHPF consists of a series passive filter and two thyristor—controlled hybrid passive filters. The new proposed filter configuration is more accuracy than conventional HPF to eliminate voltage and current harmonics. While HPF is limited to control reactive power compensation on linear and nonlinear load, AHPF supplies more precise control on it. Detailed comparison of the HPF and AHPF are given in Table 1. It can be safely said that AHPF is more accuracy and superior than HPF compared with reactive power compensation and harmonic mitigation.

**Table 1:** Comparison of the HPF and AHPF

Comparison criterions	HPF	AHPF
Voltage fed type harmonics compensating	Capable	Accuracy
Current fed type harmonics compensating	Capable	Accuracy
Reactive power compensating	Limited	Accuracy

**Fig. 6:**
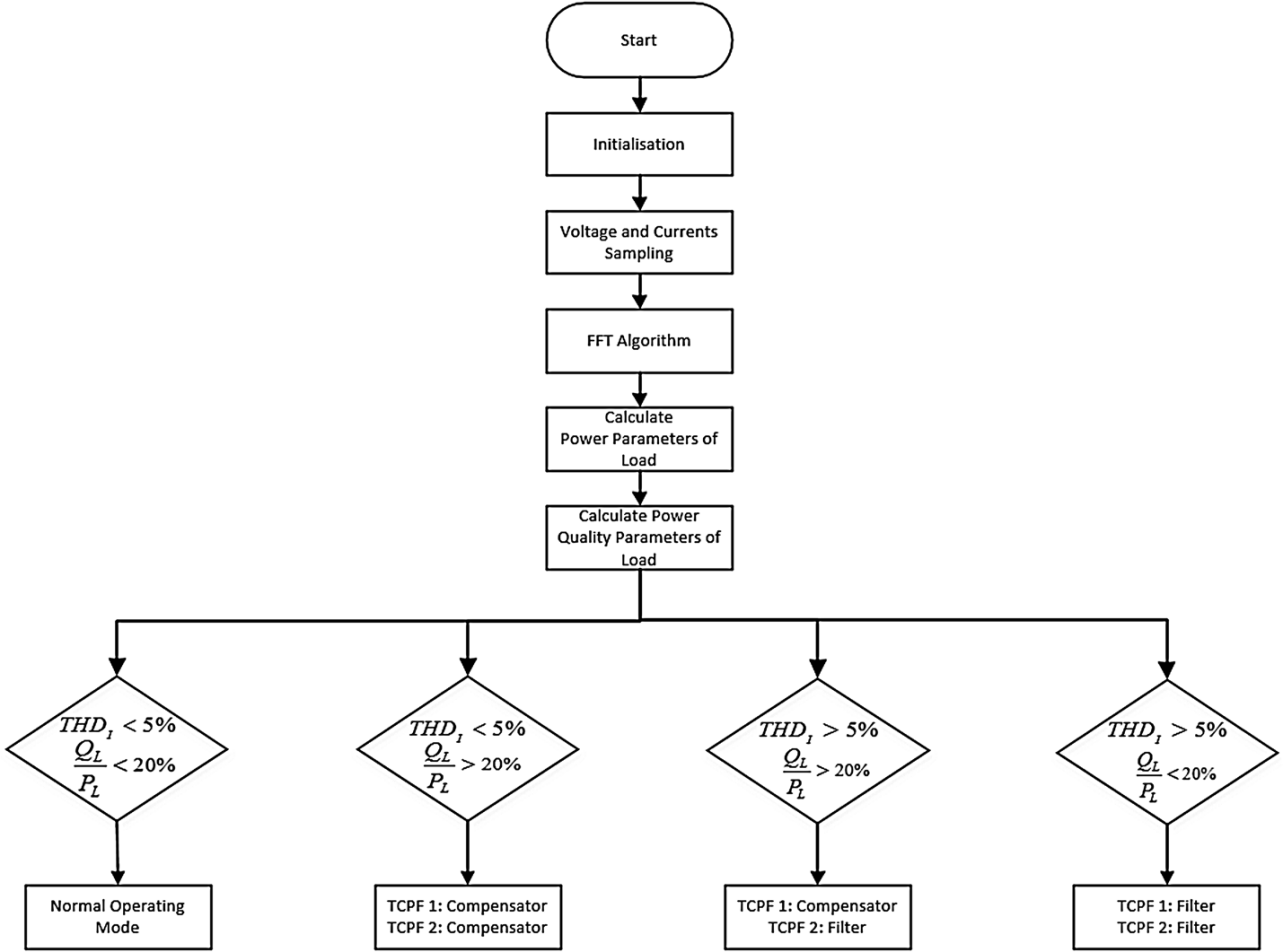
Flow chart of AHPF

Operating modes of the AHPF are listed in Table 2. Each TCPF of the proposed filter has two roles for reactive power and harmonic compensation. These roles briefly explain as compensator or filter. The roles of TCPFs in the proposed AHPF system configuration are decided by the condition of nonlinear loads. Working combinations of the TCFPs are compensator–compensator, filter–compensator, or filter–filter for harmonic mitigation and reactive power compensation. Detailed explanation of how works AHPF control system is given as flow chart in Fig. 6. In order to achieve fast and accurate power quality and power factor improvement, measurement and calculation process has to be performed precisely and accurately. Because of the non-sinusoidal form of the voltage and current sample, signal processing methods are very important to calculate the fundamental component of the power. FFT or Goertzel Algorithms are generally utilized for calculating fundamental harmonic in industrial application. Voltage and current signals at PCC and load side is sampled with 20 kHz sampling rate. Two parameters are used to decide operating modes as follows;

**Table 2:** Operating modes of the AHPF

Operating mode	TCPF 1	TCPF 2
Normal operating	–	–
Power factor improver	Compensator	Compensator
Power factor and power quality improver	Compensator	Filter
Power quality improver	Filter	Filter

- One of them is total harmonic distortion level of current (*THD*_*I*_) at the load side.
- Another one is the ratio of reactive power and active power.

If distortion level (*THD*_*I*_) at load side is smaller than 5 % and ratio of power parameters is smaller than 20 %, the AHPF is operated without compensator and filter. This operating mode is normal operating mode of AHPF. If distortion level (*THD*_*I*_) at load side is smaller than 5 % and the ratio of power parameters is greater than 20 %, the AHPF is operated as reactive power compensator. If distortion level (*THD*_*I*_) at load side is greater than 5 % and ratio of power parameters is smaller than 20 %, the AHPF is operated as harmonic filter. If distortion level (*THD*_*I*_) at load side is greater than 5 % and ratio of power parameters is greater than 20 %, the AHPF is operated as both harmonic filter and reactive power compensator at the same time.

## Simulation results

In this section, the AHPF configuration is simulated by using MATLAB/Simulink environment and Sim Power System Toolbox in order to validate the precision of the proposed configuration. For the purpose of revealing the performance of AHPF system, simulation works are also realized separately for five different parts. Nonlinear and linear load groups of simulated power system is modeled and analyzed in the first part of simulation works. In the second part, control system of TCPFs is designed by using Proportional–Integral–Derivative (PID) controller. Detailed comparison between AHPF and HPF is examined in the third part of simulation works. In the last two part of simulation works, the AHPF is simulated separately for two different scenarios in order to examine of the performance of the designed AHPF. Additionally, according to the load condition power and quality parameters of simulated power system are measured using new Simulink block that is improved for this purpose separately. The power system and simulation parameters are listed in Table 3.

**Table 3:** Power system parameters

Line voltage	*V*_*p*–*p*_	400 V
Line frequency	*f*	50 Hz
Line impedance	*L*_*s*_	0.5 mH
	*R*_*s*_	0.1 Ω
Simulation step time	*T*_*s*_	5 µs

The performance of the designed AHPF system has been simulated under current and voltage fed types of harmonic producing nonlinear loads and fixed load. Calculated values of new filter topology parameters are listed in Table 4.

**Table 4:** Calculated values of new filter topology

SPF capacitor	*C*_*SF*_	299 µF
SPF reactor	*L*_*SF*_	33.67 mH
TCPF capacitor	*C*_*PF*_	90 µF
TCPF reactor	*L*_*PF*_	113 mH

### Loads modelling

**Fig. 7:**
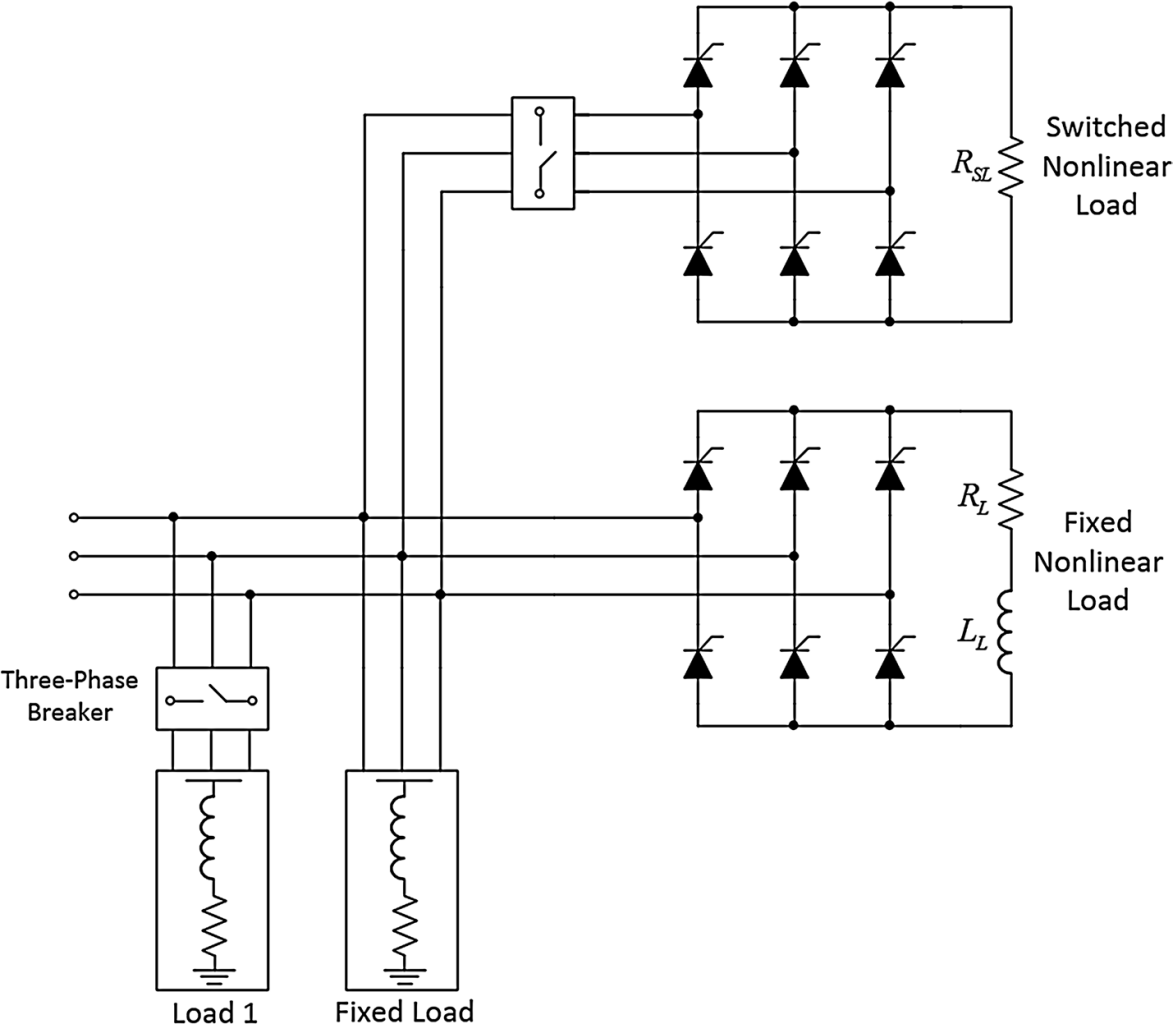
Load groups of simulated power system

Loads groups of simulation studies are modelled using two six pulse rectifiers and two fixed load groups that are made up series combination of reactor and resistor. The loads groups consist of fixed linear and nonlinear load and switchable linear and nonlinear loads. Switchable loads of simulated power system is used in order to obtain suddenly changing loads groups. Loads in simulated power system are illustrated in Fig. 7 and parameters of loads are listed in Table 5.

**Table 5:** Parameters of load models

Fixed nonlinear load	*R*_*L*_	100 Ω
	*L*_*L*_	25 mH
Switched nonlinear load	*R*_*SL*_	200 Ω
Fixed load	*P*_*FL*_	500 W
	*Q*_*FL*_	1500 VAr
Load 1	*P*_1_	100 W
	*Q*_1_	1000 VAr

**Fig. 8:**
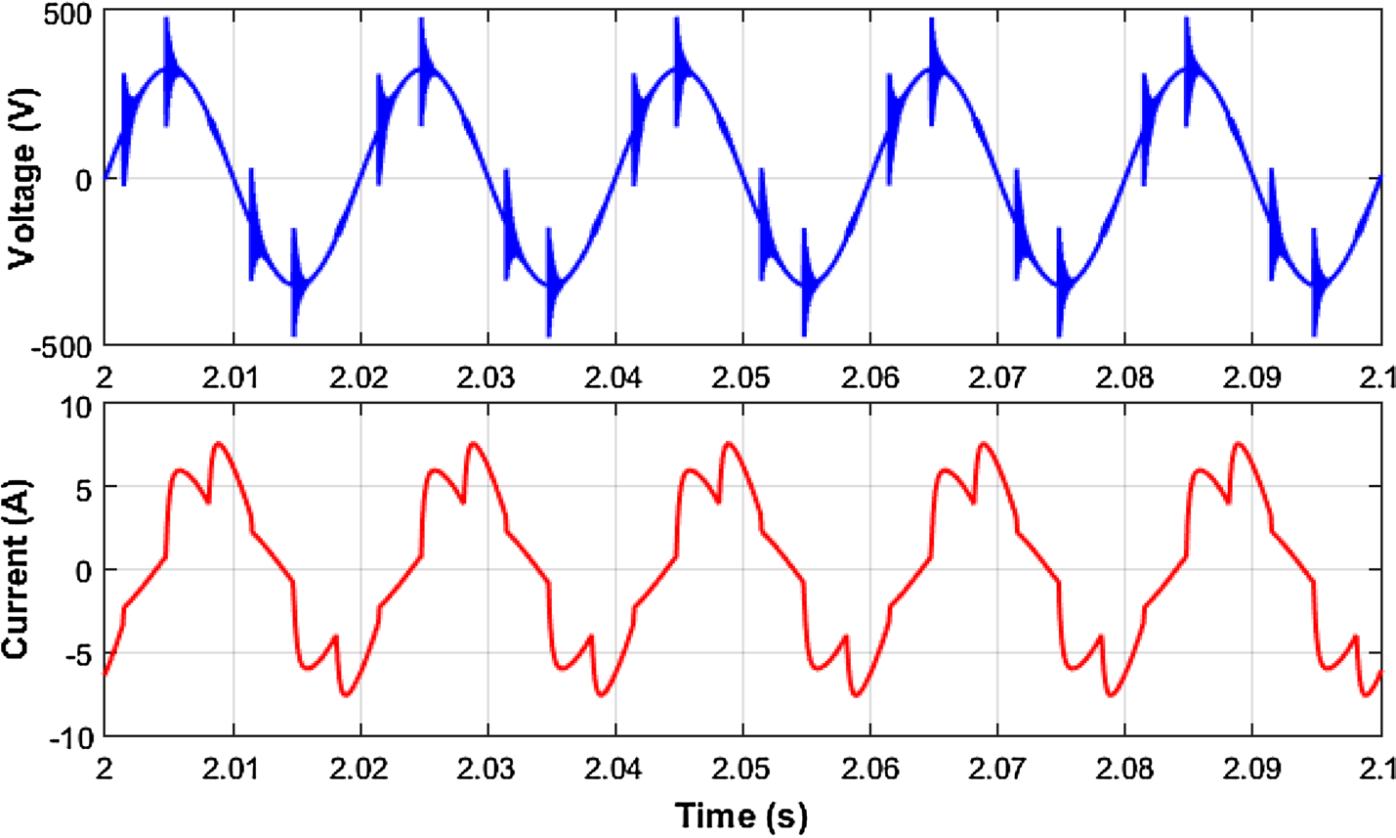
Voltage and current waveform of fixed loads

**Fig. 9:**
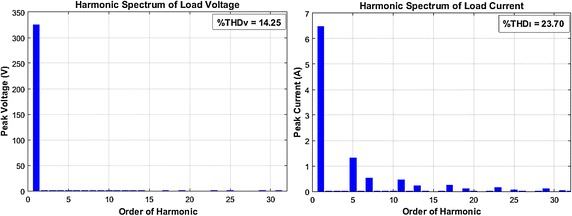
Harmonic spectrum of load current and voltage

Voltage and current waveforms of fixed loads groups is given in Fig. 8. The harmonic spectrums of single-phase voltage and current of this loads is shown in Fig. 9. The waveforms are sampled for five periods. As clearly seen in Fig. 8, voltage and current waveforms include notches and many harmonic frequencies respectively. Six pulse three phase rectifiers cause that type of distortion. As shown in Fig. 9, the load current mainly includes 5th, 7th, 9th, 11th, 13th harmonic frequencies, and total harmonic distortion of load current is 23.70 %, The harmonic distortion level of load voltage is 14.25 %.

### Control system of TCPFs

In this part, the thyristors of TCPFs are controlled by PID control system. Proportional–integral–derivative (PID) controller is one of the earlier control techniques (Åström and Hägglund 2006). Moreover, it is known that PID controller is widely used in many industrial and practical applications because of its simple structure and effective control capability (Visioli 2006). In PID controller structure, there are three coefficients such as proportional, integral, and derivative. These coefficients are summed to calculate the output of the PID controller. The control signal of the PID controller can also be expressed as below (Keel and Bhattacharyya 2008; Ang et al. 2005):

4 $$ U(t) = K_{p} e(t) + K_{i} \int {e(t)dt + K_{d} \frac{d}{dt}e(t)} $$

where the control signal *u*(*t*) is the sum of three coefficients. Each of these coefficients is a function of the tracking error *e*(*t*). The proportional (P) coefficient produces the output of controller depending on the amount of error, and the proportional coefficient increases the static accuracy and dynamic response of the system. The integral (I) coefficient reduces steady-state errors through low-frequency compensation. The derivative coefficient improves transient response through high-frequency compensation. Each of these coefficients operates independently of each other (Åström and Hägglund 2006; Silva et al. 2002).

**Fig. 10:**
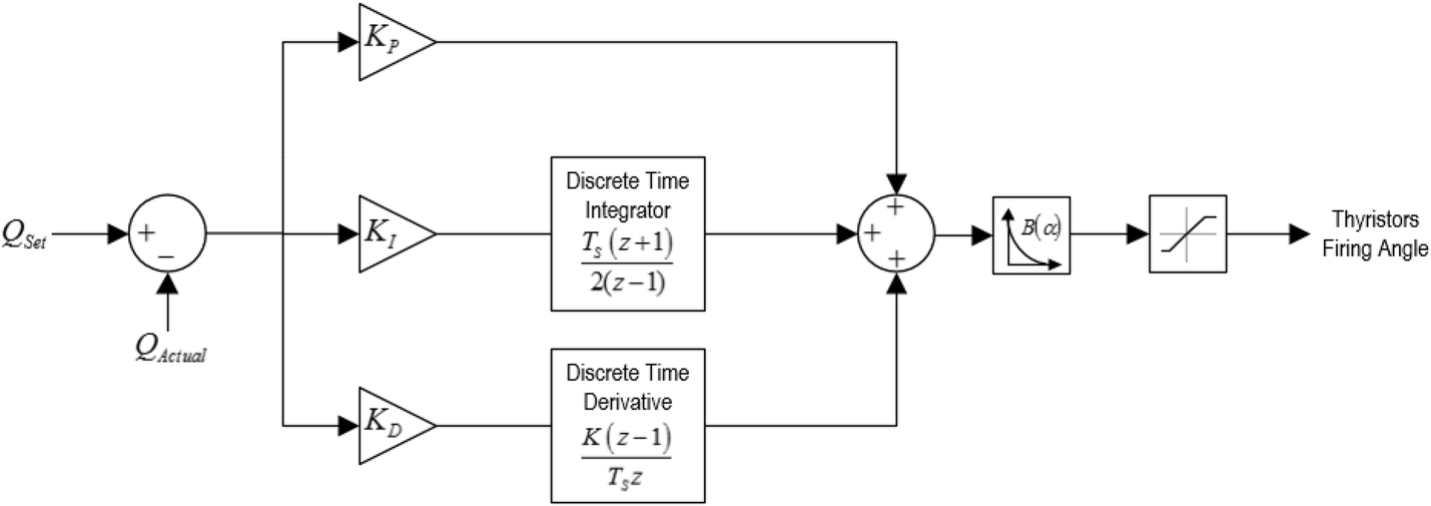
Block diagram of control system

The block diagram of PID control system for TCPF is given in Fig. 10. Proportional, integral and derivative parameters of the control system are listed in Table 6.

**Table 6:** Parameters of control system

Proportional gain	*K*_*P*_	0.3
Integral gain	*K*_*I*_	35
Derivative gain	*K*_*D*_	0.02

### Comparison of conventional HPF and AHPF

In this part of simulation studies, proposed AHPF configuration is compared to conventional HPF. The effect of proposed configuration on the simulated power system is examined for reactive power compensation and harmonic mitigation. Although HPF has only filter mode, the AHPF has three different operating modes. While HPF is only operated power quality improver, AHPF is operated both power factor and power quality improver in this simulation studies.

**Fig. 11:**
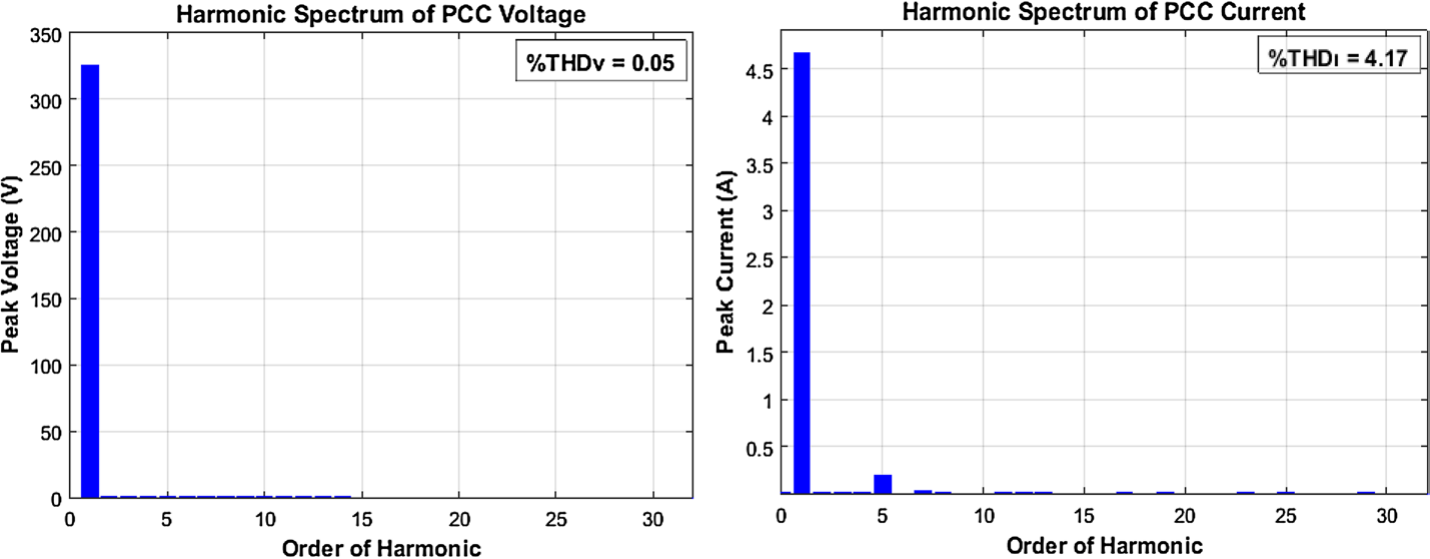
Harmonic spectrum of voltage and current at the PCC side with HPF

Firstly, fixed linear and nonlinear loads groups are used in order to analyze harmonic mitigation performance of HPF and AHPF. Voltage and current type of harmonics of the loads groups are compensated with HPF and AHPF configurations, respectively. Harmonic spectrums of voltage and current at the PCC side with HPF configuration are given in Fig. 11.

As shown in Fig. 11, total harmonic distortion levels of voltage (*THD*_*V*_) and current (*THD*_*I*_) is 0.05 and 4.17 %, respectively. THD level of current (*THD*_*I*_) is smaller than specific limit that is mentioned IEEE 519-1992 standard. As a result, conventional HPF configuration is successful in compensating voltage and current types of harmonics at load side.

**Fig. 12:**
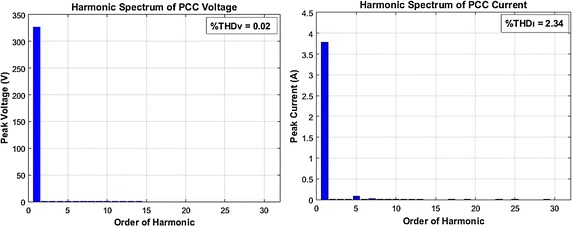
Harmonic spectrum of voltage and current at the PCC side with AHPF

**Fig. 13:**
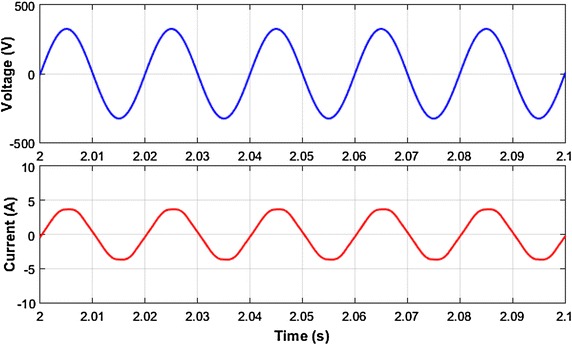
Voltage and current waveform at the PCC side with AHPF

Harmonic spectrums of voltage and current at the PCC side obtained by AHPF are given in Fig. 12. Waveforms of current and voltage at the PCC side measured by AHPF are illustrated in Fig. 13.

As shown in Fig. 12, THD level of PCC voltage (*THD*_*V*_) is nearly close to 0.00 and THD level of PCC current (*THD*_*I*_) is 2.34 %. As clearly seen in this Fig. 13, the voltage and current waveforms at the PCC become close to a sinusoidal form after using by AHPF. It is explicitly illustrated from Figs. 12 and 13 that proposed filter configuration almost completely eliminate the harmonics caused by nonlinear load. As shown in Fig. 13, due to AHPF is capable of working reactive power compensation mode, current of loads at the PCC is decreased compared to unfiltered conditions. Consequently, it is observed that AHPF is more precise than HPF.

**Fig. 14:**
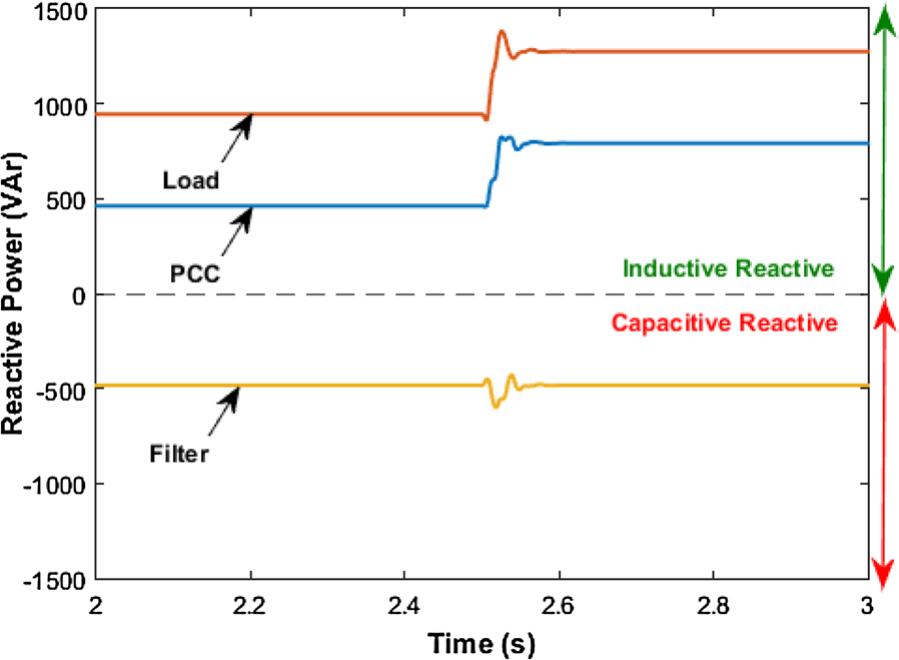
Reactive power at the PCC and load side with HPF

Lastly, comparison of reactive power compensation performances of HPF and AHPF are studied in this part of simulation. For this purpose, sudden switchable linear load is used for analyzing performances of reactive power compensation. Total simulation time is 3 s. Load 1 is switched on 2.5th seconds in simulation time. Reactive power at PCC and load side with HPF is shown in Fig. 14.

After the switched on load 1, it is observed that reactive power of loads groups has increased from 945 to 1276 VAr. As clearly seen in Fig. 14, reactive power compensation performance of HPF is limited and fixed. Capacitive reactive power of the HPF is 483 VAr. Before switched on load 1, power factor value at PCC side is 0.79. After switched on load 1, it is shown that power factor has decreased. Reactive power at the PCC side has increased from 462 to 793 VAr. Performance of HPF configuration is not satisfactory for reactive power compensation.

**Fig. 15:**
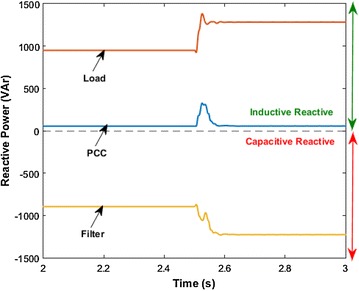
Reactive power at the PCC and load side with AHPF

Reactive power compensation performance of AHPF is shown in Fig. 15. In this simulation parts, reactive power at the load side has increased from 945 to 1276 VAr by means of switching on load 1. Respond to it, AHPF has supplied variable capacitive reactive power. Supplied capacitive reactive power is increased from 896 to 1226 VAr after the 2.5th seconds. Power factor at the PCC side is fixed 1.00 before and after switched on load 1. It is explicitly illustrated from Fig. 15 that AHPF provides more accuracy than HPF for reactive power compensation.

### Scenario 1: Switching load 1

**Fig. 16:**
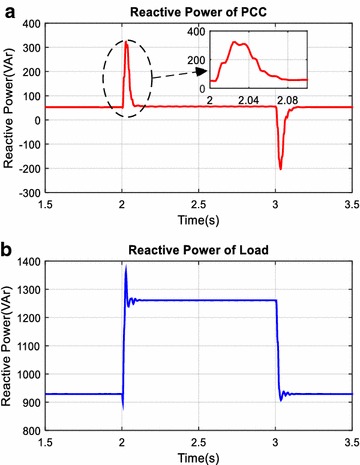
Reactive power at PCC (**a**) and load side (**b**)

**Fig. 17:**
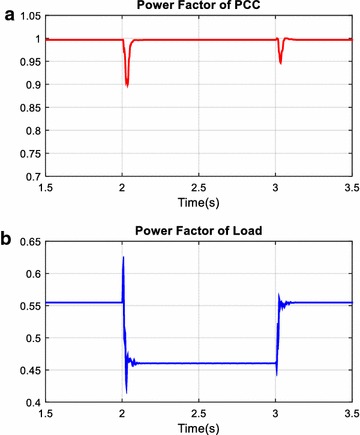
Power factor of PCC (**a**) and load (**b**)

The unexpected events in power systems such as suddenly switched on/off nonlinear and linear loads cause disturbance effects on filter, reactive power compensator, and power systems. In this simulation scenario, performance of AHPF is examined against abovementioned disturbance effects. Total simulation time is 4 s. In addition to fixed loads, the load 1 is switched on 2th seconds and switched off 3th seconds in simulation time. Reactive power and power factor changing at the PCC and load side are given in Figs. 16 and 17 and are plotted red and blue colors on all figures respectively. As shown in Fig. 16, reactive power value at the load side has increased from 930 to 1260 VAr after 2th seconds. In response to this changing, reactive power value at the PCC has suddenly increased from 55 to 310 VAr. However, it has rapidly reached set value of reactive power for PCC side by means of PID controller. The settling time of control system is 0.08 s and it is acceptable for reactive power compensation. As clearly seen in Fig. 17, although power factor value at the load side is decreased from 0.55 to 0.46 after 2th seconds, power factor value at the PCC side has fixed 0.99.

**Fig. 18:**
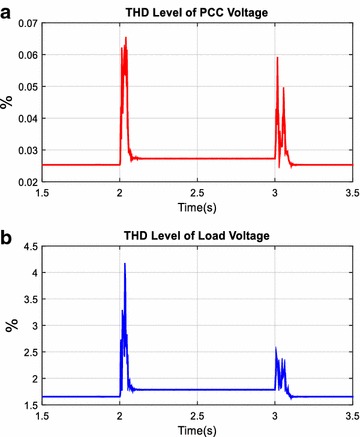
THD level of voltage at PCC (**a**) and load (**b**)

**Fig. 19:**
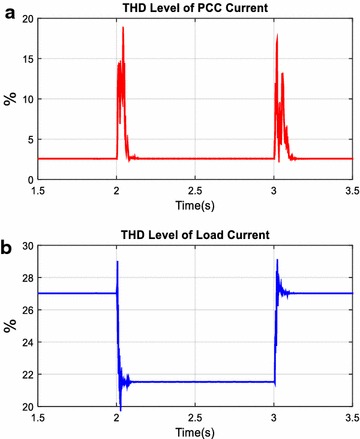
THD level of current at PCC (**a**) and load (**b**)

Total harmonic distortion level of voltage (*THD*_*V*_) and current (*THD*_*I*_) with AHPF at the load and PCC side are shown in Figs. 18 and 19. As clearly seen in Figs. 18 and 19, while *THD*_*V*_ value at the load side has increased from 1.6 to 1.9 %, *THD*_*V*_ value at the PCC side has fixed after switched on load 1. Although THD level of current at the load side has decreased from 27.02 to 21.52 %, THD level of current at the PCC side has fixed to 2.56 % after switched on load 1. Consequently, before and after switched on load 1, *THD*_*V*_ and *THD*_*I*_ values at the PCC are smaller than specific limit and the AHPF is smoothly compensated harmonics and reactive power for this simulation scenario.

### Scenario 2: Switching nonlinear load

**Fig. 20:**
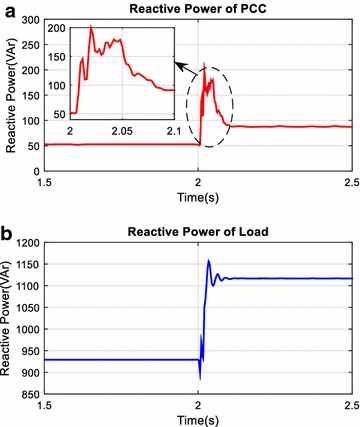
Reactive power at PCC (**a**) and load side (**b**)

**Fig. 21:**
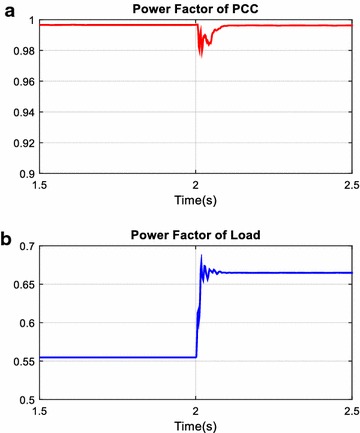
Power factor of PCC (**a**) and load (**b**)

This part of simulation studies is performed to examine harmonic mitigation and reactive power compensation performance of AHPF. For this purpose, in addition to fixed load groups a new nonlinear load is suddenly switched on 2th seconds. Reactive power and power factor changing at the PCC and load side are given in Figs. 20 and 21 and are plotted red and blue colors on all figures respectively. As it is also clearly seen in this Fig. 20 that reactive power value at the load side has increased from 930 to 1110 VAr after 2th seconds. However, reactive power value at the PCC side has increased from 50 to 87 VAr and power factor value at the PCC side is nearly fixed to 1.00.

**Fig. 22:**
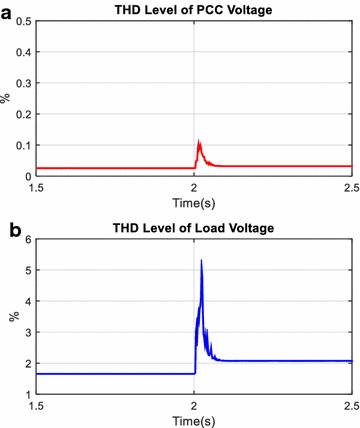
THD level of voltage at PCC (**a**) and load (**b**)

**Fig. 23:**
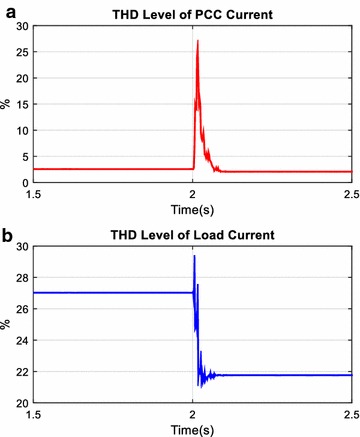
THD level of current at PCC (**a**) and load (**b**)

The total harmonic distortion level of voltage (*THD*_*V*_) and current (*THD*_*I*_) with AHPF at the load and PCC side are shown respectively in Figs. 22 and 23. Before the 2th seconds, while *THD*_*V*_ value at the load side is 1.65 %, *THD*_*V*_ value at the PCC side is 0.02 %. After the 2th seconds, *THD*_*V*_ value at the load side has increased 2.07 %. However, THD level of voltage at the PCC side has not changed. As clearly seen in Fig. 23, although *THD*_*I*_ value at the load side has decreased from 27.02 to 21.77 % after the switched on the new nonlinear load, *THD*_*I*_ value at the PCC side has decreased from 2.6 to 2.0 %. As a result of this scenario, it is observed that AHPF is so useful for power quality and power factor improvement.

## Conclusion

In this paper, a new hybrid passive filter that is named AHPF is developed in order to both harmonic mitigation and reactive power compensation. Reactive power and harmonic compensation performance of AHPF compared to conventional HPF. Many simulation studies have been performed for this purpose. The mainly advantage of proposed filter, while HPF provides limited capacitive reactive power, AHPF provides precise capacitive reactive power for power factor improvement. Whole simulation studies show that, THD levels of current and voltage at the PCC side are acceptable for the power quality standards. Additionally, simulation results indicate that power factor of the system is fixed about 1.0 for all simulation conditions. As a result of this studies, performance of AHPF configuration is more accuracy in order to reactive power compensation and harmonics mitigation as compared to conventional HPF.

## Abbreviations

AHPF: advanced hybrid passive filter; HPF: hybrid passive filter; TCPF: thyristor controlled parallel passive filter; SPF: series passive filter; THD: total harmonic distortion; PCC: point of common coupling.

### List of symbols

*Z*: impedance
*L*: inductor
*C*: capacitor
*V*: voltage
*Q*: reactive power
*P*: active power
*f*: frequency
*T*: time
*R*: resistor
*K*: gain

### Subscripts

*S*: source
*SF*: series passive filter
*PF*: parallel passive filter
*L*: load
*I*: current
*v*: voltage
*p*–*p*: phase–phase
*SL*: switched load
